# CoagVDb: a comprehensive database for coagulation factors and their associated SAPs

**DOI:** 10.1186/s40659-015-0028-5

**Published:** 2015-07-19

**Authors:** Shabana Kouser Ali, C George Priya Doss, D Thirumal Kumar, Hailong Zhu

**Affiliations:** Medical Biotechnology Division, School of Biosciences and Technology, VIT University, Vellore, Tamil Nadu 632014 India; Department of Computer Sciences, Hong Kong Baptist University, Kowloon Tong, Hong Kong

**Keywords:** Coagulation cascade pathway, Coagulation factor proteins, Single amino acid substitutions, SIFT, PolyPhen 2, I-Mutant 3, Fathmm, Align GVGD, PhD-SNP, SNPs&GO, SNAP

## Abstract

The current state of the art in medical genetics is to identify and classify the functional (deleterious) or non-functional (neutral) single amino acid substitutions (SAPs), also known as non-synonymous SNPs (nsSNPs). The primary goal is to elucidate the mechanisms through which functional SAPs exert their effects, and ultimately interrogating this information for association with complex phenotypes. This work focuses on coagulation factors involved in the coagulation cascade pathway which plays a vital role in the maintenance of homeostasis in the human system. We developed an integrated coagulation variation database, CoagVDb, which makes use of the biological information from various public databases such as NCBI, OMIM, UniProt, PDB and SAPs (rsIDs/variant). CoagVDb enriched with computational prediction scores classify SAPs as either deleterious or tolerated. Also, various other properties are incorporated such as amino acid composition, secondary structure elements, solvent accessibility, ordered/disordered regions, conservation, and the presence of disulfide bonds. This specialized database provides integration of various prediction scores from different computational methods along with gene, protein, and disease information. We hope our database will act as a useful reference resource for hematologists to reveal protein structure–function relationship and disease genotype–phenotype correlation.

**Electronic supplementary material:**

The online version of this article (doi:10.1186/s40659-015-0028-5) contains supplementary material, which is available to authorized users.

## Background

The mechanism of blood coagulation plays a central role in the maintenance of homeostasis in the human system. Three major interrelated constituents of the hemostatic pathway include platelets, endothelium, and coagulation proteins which maintain fluidity of the blood in the normal state and also arrests bleeding by blood clot formation. The coagulation system is necessary for containing the blood loss from a vascular injury due to trauma results in fibrin formation and platelet activation. Blood coagulation factors function in an incessant cascade to achieve one central goal, which is the arrest of bleeding. Even miniature change in the structure or concentration of these factors in the blood can disrupt the entire system of blood clotting. A defect in the normal blood clotting mechanism (reduced or absence of coagulation proteins) leads to several types of bleeding disorders which are highly prevalent in global populations (Additional file 1: Table S1). Among them, Von Willebrand disease is the most common inherited bleeding disorder that affects nearly 1% of the world’s population [1]. Coagulation system in human is initiated by two different mechanisms i.e. the process of contact activation (intrinsic) and the action of tissue factor (extrinsic). These two separate pathways converge and activate the final common pathway leading to fibrin formation. The two pathways occur in a cascade involving the action of twelve different factors each working to activate the successive factors.

A recent development in high throughput screening methods and cost reduction genotyping has generated a tremendous amount of information about the existence of an association between single amino acid polymorphisms (SAPs) and bleeding and coagulation disorders. These advancements have yielded a considerable quantity of information especially on genomic variant data (SAPs) and revolutionized the current field of biology and medicine, i.e., personalized medicine. The current state of art in clinical genetics is to identify and classify the functional (deleterious) or non-functional (neutral) SAPs in diseases, to elucidate the mechanisms through which functional SAPs exert their effects [2, 3]. SAPs also known as nonsynonymous SNPs (nsSNPs); result in a change of amino acid sequence that can alter the protein function. SAPs can be categorized into harmful (deleterious) and neutral. Most of the SAPs are not harmful [4]. Half of all genetic changes related to human diseases are associated to SAPs [5]. Therefore, discriminating the harmful from neutral ones, from a pool of millions of SAPs, remains as a considerable challenge in mutational research [2, 3, 6, 7]. This categorization can assist in better understanding of the genotype/phenotype relationship and drug response to disease. Several large repository databases are made available through World Wide Web in providing information regarding millions of SAPs such as dbSNP [8], Ensembl [9] and UniProt [10]. Scientists are facing a major challenge in the identification, functional characterization, and the association between SAPs and disease susceptibility in the large-scale analysis.

Recent technological advances resulted in the accumulation of an extensive amount of information on each component of this complex coagulation mechanism. An increase in the occurrence of heritable deficiencies of the blood clotting factors has led to development of few reliable sources, such as ClotBase (http://www.clotbase.bicnirrh.res.in/) [11], Factor VIII variation database (http://www.factorviii-db.org/) [12], factor IX variation database (http://www.factorix.org/) [13], and VWFDb (http://www.vwf.group.shef.ac.uk/) [14] to provide information on the sequence, structure and phenotypes of various coagulation proteins. In this paper, we propose a user-friendly and freely accessible interface Coagulation Variation Database (CoagVDb), which allows the user to collect the sequence and variant information regarding the proteins involved in coagulation cascade pathway. The primary goal of the constructed CoagVDb is intended to integrate most reliable biological information about the genes, proteins and variants involved in coagulation cascade pathway. Moreover, we captured the computational prediction scores of SIFT [15], PolyPhen 2 [16], I-Mutant 3 [17], fathmm [18], Align GVGD [19], PhD-SNP [20], SNPs&GO [21] and SNAP [22] to classify SAPs as deleterious/harmful or tolerated/neutral. Development of new techniques and production of efficient drugs for use against bleeding disorders requires information from protein structure as well. The information derived from primary sequence properties (physicochemical properties of the amino acids) is essential for exploring the effects of each SAP on protein structure and function. Based on this, we have incorporated information about three dimensional structures, amino acid composition, conserved regions, disulfide bonds, ordered/disordered regions, secondary structure elements, and solvent accessibility in the constructed database. Studying the SAPs and their effects along with sequence analysis information is often crucial for understanding the effects on protein functionality and biochemical reactions. Analyzing and understanding structure–function relation of the associated deleterious SAPs for a particular coagulation factor protein will open new possibilities for diagnostic and therapeutic efforts.

To best of our knowledge, there is no comprehensive database capturing the protein *sequence, structure and* SAPs information along with the in silico prediction scores for coagulation factors in one platform. The goal of this project is aimed to design and implement a freely accessible web interface CoagVDb that collates the genomic information in a curated, yearly updated, concise and well-structured manner. We hope this platform will help researchers in understanding the genotype-phenotype relationship, which is the first and foremost important step in drug research and development. This integrated multifunctional reference resource is freely available at http://www.info.vit.ac.in/CoagVdb/index.html.

## Results

### Search for gene symbol

The CoagVDb provides access to genomic information related to coagulation factors. Information consists of HGNC gene name, gene symbol, gene ID, organism source, taxonomy identification, chromosome number, chromosome location, chromosome sequence (NC), and NCBI map viewer. Gene related information present in other databases such as OMIM, Ensembl, and UniProtKB were also interlinked. This allows the user to search for Ensembl gene ID, HUGO gene name or HGNC gene ID, Entrez Gene ID, and OMIM gene ID. The gene information such as the DNA sequence and chromosomal location was obtained from NCBI. Epigenetic information such as histone modifications, chemical changes in DNA, chromatin accessibility, gene expression and small RNA expression is available in the epigenomics section of the NCBI database. Further, to enrich the available information on the gene sequence, the CoagVDb provides integration with the NCBI MapViewer in the form of hyperlinks. Moreover, the database forms a simple network between various other databases and information sources through conveniently openable links in a new tab. Literature information on the gene coding the factors, properties of the proteins involved in the mechanism, and the consequences of variations that result in disease phenotypes were obtained from published articles available in PubMed, OMIM and UniProtKB and referred with PubMed ID. Some of the information integrated with the other database includes HGNC, Entrez, Ensembl, UCSC, OMIM, UniProtKB, UniGene, RefSeq, and KEGG.

### Search by variant name

To display the records of 3187 SAPs, we designed user-friendly web interface in CoagVDb. To increase the accuracy of SAPs annotation, we initially collected the SAPs related information from dbSNP and overlapped with the variant information from UniProt. We listed the SAPs with reference links to rsIDs/variants, amino acid position, allele change, contig position, protein ID (FASTA sequence identification number (NP) and UniProt sequence number). The amino acid change in each variant is represented by wild and new residue after mutation (Single letter amino acid code) e.g. WT+POS+NEW.

### Protein information

Information related to the protein can be accessed in two ways; sequence and structure information.

#### Sequence information

This section of the database entry contains information about the protein sequence that integrates sequence analysis information from various computational methods. The sequence information includes sequence length, amino acid composition, solvent accessibility, secondary structural elements, ordered/disordered regions, cysteine residue location, disulfide bond formation and conservation score provided in the form of a table.

#### Amino acid composition

Amino acid sequence composition analysis can provide the most direct information about the functional mutation sites of the protein. Recent studies have explored the occurrence of various amino acids along with their biophysical characteristic in the native and mutant state of numerous proteins [23–25]. We calculated the composition of each amino acid in corresponding coagulation factor protein sequence by Statistical Analysis of Protein Sequences [26]. For this analysis, we submitted the individual protein FASTA sequence as an input file.

#### Secondary structure analysis and solvent accessibility

We analyzed the occurrence, location and distribution of secondary structural elements, α-helices, β-strands, turns, and bends. Amino acids distributions among these elements were considered to be the essential structural components of protein scaffolds. Secondary structure and solvent accessible area of each amino acid in the protein sequence was calculated using NetSurfP ver. 1.1 **[**27]. The secondary structure elements were represented as H: Alpha-helix; G: 3-10-Helix; I: Pi-helix (extremely rare); E: Extended strand; B: Beta-bridge; T: turn; S: Bend; and C: The Rest. Solvent accessible area of each amino acid is classified as buried and exposed and represented in red and black color, respectively.

#### Disordered residues

The disordered region in a protein sequence is characterized by the presence of enriched polar and charged amino acids with low percentage of hydrophobic amino acids [28]. DISpro [29] was utilized to predict the probability of each amino acid residue to be ordered or disordered. The residues were designated as O-Ordered; D-Disordered in the output file.

#### Cysteine residues and disulfide bonds

Studies have highlighted the importance of Cys residues and disulfide bonds in protein folding [30]. Amino acid residue change to (or) from Cys is most likely to destabilize a protein structure. Taking into consideration, we extended our analysis of sequence information by the application of DIpro [31] to predict disulfide bonds and estimate the number of disulfide bonds in a given protein sequence.

#### Sequence conservation

Disease-causing SAPs often reside in highly conserved positions. Assessment of non-neutral SAPs is primarily based on phylogenetic information (i.e. correlation with residue conservation) extended to an individual scale with structural approaches. A multiple sequence alignment of the homologous sequence reveals the position at which amino acids are conserved throughout evolutionary time. These positions can be critical for protein function [32]. Initially, we performed multiple sequence alignments (MSA) using multiple sequence comparison by log-expectation (MUSCLE), a web-based tool to align multiple sequences from several vertebrate species including humans [33]. We searched the protein sequence of coagulation factors against a sequence database to find sequences of homologous proteins. The importance of a residue for maintaining the structure and function of a protein can usually be inferred based on the conservation pattern. ConSurf [34] quantifies the degree of conservation at each aligned position to represent localized evolution. This server provides the evolutionary conservation profiles of protein or nucleic acid sequence or structure by first identifying the conserved positions using MSA and then calculates the evolutionary conservation rate using an empirical Bayesian inference.

#### Structure information

Data on the available three-dimensional (3D) structure coordinates of coagulation factor proteins were listed in this database section. Experimentally determined structures either by X-ray or NMR were obtained from the protein data bank (PDB) [35]. In addition, we incorporated 3D structure resolution, chain type, and amino acid residue position information.

### Prediction tools

**Figure 1:**
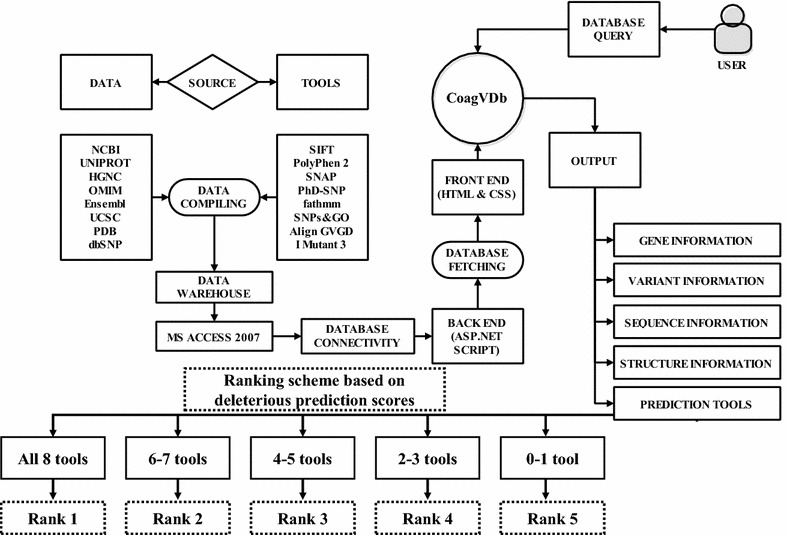
Coagulation variation database (CoagVDb) construction.

We predicted the functional effect of each SAP as pathogenic/deleterious or neutral/tolerated by using computational prediction methods such as SIFT [15], PolyPhen 2 [16], I-Mutant 3 [17], fathmm [18], Align GVGD [19], PhD-SNP [20], SNPs&GO [21] and SNAP [22]. The methods mentioned above utilize different input features in making their predictions, but the ultimate goal is to discriminate deleterious or functional SAPs from neutral ones. We submitted either gene identification (GI) number or FASTA sequence or Swiss-Prot protein code, substitution position (sequence residue number) and native or wild type residue (Single letter amino acid code) and new residue after mutation as mutant (single letter amino acid code) e.g., WT+POS+NEW as input. Integrating the prediction scores of sequence (SIFT, PhD-SNP, Align GVGD, and fathmm) and combination of sequence and or structure based (PolyPhen-2, SNAP, SNPs&GO and I-Mutant 3) computational methods may provide wider coverage and more accurate predictions in the study of SAPs. Above utilized methods derive their information from the multiple sequence alignment of the homologous sequences to give more information about the extent of conservation based on the input generated internally (SIFT & SNPs&GO) or submitted by the user (PolyPhen-2 & Align GVGD). Detailed information regarding the prediction scores of the above eight computational methods is described in Additional file 1: Table S2. We have introduced a ranking scheme to prioritize the variants based on the prediction score designated as ‘deleterious’ obtained from the above eight computational methods. Variants/rsIDs showing all 8 tool prediction score as deleterious will be ranked as ‘1’, variants showing 6–7 tools prediction score as deleterious will be ranked as ‘2’, variants showing 4–5 tools prediction score as deleterious will be ranked as ‘3’, variants showing 2–3 tools prediction score as deleterious will be ranked as ‘4’, variants showing 0–1 tool prediction score as deleterious will be ranked as ‘5’ respectively (Figure 1).

### Web interface

The freely accessible CoagVDb allow users to perform ‘quick search’ using keyword gene symbol, variant/rsIDs in the left navigation bar. We have listed out the additional information regarding the genes, disease, diagnosis, FASTA sequence, download, and site map of 29 coagulation proteins. Gene button module allows the user to provide a direct link to 29 gene information. Disease button allows user to access the useful information such as disease name, inheritance pattern, OMIM ID, disease classification (primary and secondary hemostasis) and occurrence (most common, less frequent and extremely rare). Diagnosis button provides information about the preliminary screening protocol, laboratory evaluation of coagulation disorders of common and multiple pathways and diagnosis of coagulation disorders using preliminary screening tests. Download button allow the user to download the gene, protein, variant, tool prediction related information of 29 coagulation protein in.xls format. Lastly site map was created to provide an overview of the database which allow user to access and navigate in a friendly manner. In the main web interface section ‘home’, we have provided the diagrammatic representation of coagulation cascade pathway which illustrates the involvement of various factors in intrinsic, extrinsic and common pathways. The active form of coagulation factors is represented in the grey color oval button with a hyperlink, whereas orange, violet, green color oval button represents the inactive form of factors in intrinsic, extrinsic and common pathways. The main taskbar “search” button allows the user to pick up gene, variant, sequence, structure, and prediction tools information. Clicking on the information tab enables the user to display the detailed information page of the corresponding entries with a hyperlink in the browser. In order to make an easy way to jump between sections, we have provided the all the information regarding a gene i.e. variant, sequence, structure, and prediction tools information on the same page. In addition, we have provided ‘Resource’ module which allows user to cross-link to other databases (NCBI, OMIM, HGMD, etc.), computational prediction methods (SIFT, PolyPhen2, PHD-SNP etc.) and related links (related coagulation factor databases (ClotBase [11], Factor IX Mutation Database [36] etc.) with their corresponding hyperlinks. Lastly, help button provides guidance to user how to access different search fields using PLAU as an example.

### Resources

In the navigation bar resource section, we have listed database, tool information, and their related links. Database tab lists out the biological database source which is available online along with their hyperlinks. Tool information tab provides the detailed information about the computational methods which are employed to classify SAPs. Lastly, related links tab contains the information about the existing databases related to coagulation factors.

### Comparison to existing databases

To the best of our knowledge related to coagulation factors, only a few databases are available online during the construction of this database. Most of the existing online available databases ClotBase [11], Factor VIII variation database [12], factor IX variation database [13], VWFDb [14], and FXI Deficiency Mutation Database [37] are centered towards individual coagulation factors. In comparison, ClotBase [11] offers compiled data on the blood coagulation proteins. Information regarding the change in amino acid sequence, evolutionary conserved regions, mutations, and other curated data has been made available to this database. Von Willebrand Factor database or VWFdb is an online database that centers on von Willebrand disease. This database primarily contains sequence variants data and provides additional resources to understand the disease association. The Haemophilia A Mutation, search, test, and resource site (HAMSTeRS) was initiated in 1996. This contains information on factor VIII of blood coagulation and extensive data on the point mutations, insertions, and deletions. Data obtained from computational analysis of the mutations and structural studies have also been included. Now this has been shifted to UCL F8 DB (HADB/HAMSTeRS). CoagMDB is a database that carries information on five serine protease factors of the blood coagulation pathway. This interactive database incorporates all five factors factor II, factor VII, factor IX, factor X and protein C and their corresponding mutational information. The mutations were correlated with experimentally quantifiable phenotypes with the help of data available on consensus domain structures. The FXI Deficiency Mutation Database was created to concentrate the information available regarding the mutations in the gene sequence of factor XI.

In comparison to the databases mentioned above, the information available in CoagVDb spans out in following ways: first, a simplified platform for viewing all coagulation factors along with gene/protein and rsIDs/variant information. It links HGNC, Entrez, Ensembl, UCSC, OMIM, UniProtKB, UniGene, RefSeq, and KEGG. Secondly, we have provided sequence information (amino acid sequence length, composition, solvent accessibility, secondary structural elements, ordered/disordered regions, cysteine residue location, disulphide bond formation and conservation) along with available 3D structure information (X-ray or NMR). This feature will allow users to access the physicochemical characteristic of each native and mutant amino acid. Third, we have included pathogenicity prediction scores for each SAP using various sequence and structure based prediction methods will allow the user to discriminate deleterious SAPs from neutral ones from a pool. Lastly, we have applied a ranking scheme to prioritize the functional SAPs based on deleterious scores obtained from the computational prediction methods. This added advantage over the existing databases efficiently helps to identify and classify the SAPs that alter the function of coagulation factor proteins. Moreover, rich content made available in CoagVDb is easy to use and interpret by any end user.

## Discussion

A recent survey states that SAPs constitute more than 50% of the known mutations are involved in human individual Mendelian diseases [38]. It is also estimated approximately that each may hold 24,000–40,000 SAPs and most of them found to be deleterious [39]. Most of the SAPs lack experimental annotation of their functional impact. Differentiating functional prediction of an SAP being deleterious (significant phenotypic consequences) from neutral one (without phenotypic change) is of prime importance in understanding the genetic basis of the disease. This discrimination and prediction of the phenotypic effect of a genomic pool remains as a major challenge for experimental biologists due to laborious and time-consuming process involved. Alternatively, computational methods can discriminate functionally deleterious SAPs from non-deleterious ones with significant accuracy while being relatively fast. They classify the SAPs as deleterious or neutral based on the physicochemical properties of amino acids in sequence or structure context. Numerous methods have been proposed online and classified as sequence-based or structure-based methods. They utilize evolutionary sequence conservation, structure information, and the combination of sequence and structure information in making their predictions. Sequence-based methods (SIFT, fathmm, and PhD-SNP) have a added advantage over the structure-based methods (PolyPhen 2, SNAP, and SNPs&GO) in making their predictions, as they can be applied to any proteins with known relatives. Similarly, structure-based methods incorporate the physicochemical properties of amino acids along with known 3D structures to make their predictions. Because of the usage of different algorithms in making their prediction, each method has its strength and weakness. The results obtained from the comparative studies [40–45] indicate that the use of combination of prediction methods with sequence and structure information may provide wider coverage and more accurate way for SAP analysis. In this context, we incorporated the prediction scores of well-known sequence and structure-based methods in CoagVDb. The uniqueness of the developed database is represented by three level of data integration. First is to connect all the related biological information such as a gene, protein, variant (SAPs) and published literature of the coagulation factors that are involved in the complex coagulation cascade pathway. Second is to provide primary sequence and structure information. Third is to identify and discriminate functionally deleterious SAPs from neutral ones in coagulation cascade proteins. This will provide a way to filter the SAPs, thereby leading to a better selection of SAPs to be included in further genotyping.

## Conclusion and future update

We conclude that this constructed database will be of great aid to clinicians in applying a biological prioritization strategy when selecting an SAP for further analysis. This will allow building relationship between the disease-related mutations and structural properties of proteins. The inclusion of online submission facility will keep the database up-to-date. In future, we would like to expand the database by including a comprehensive information on 3′ and 5′ UTR SNP information along with sequence accession numbers (NM). In this step, we will characterize the functional significance of each regulatory SNP by using various computational methods. Lastly, we will also include the 3D model of the mutant proteins.

## Methods

The primary objective of CoagVDb is to combine various biological information components that are involved in blood coagulation cascade. CoagVDb hosts a colossal amount of useful data on coagulation factors and their associated variants. In this section, we describe the entire process of database construction, data generation, and structuring of the database.

### Database construction

The biological information regarding coagulation factors is scattered in many public domains and also cross-linked with other databases [11–14]. Till date, there is no straightforward method to retrieve all the relevant biological information regarding coagulation factors in one platform. CoagVDb offers all relevant information on genes, proteins and SAPs that are associated with bleeding and coagulation disorders. Figure 1 illustrates the construction of the database. Major front end software components empowered in the database includes Html 5.0, ASP.NET, and Microsoft Visual Studio 2010. The data information exported from the database is in Html 5.0 and CS script format which allow users to display supplementary information in a different web page. The web interface is designed using the interface ASP.NET and back-end data constructed using MS access 7.0. (Version 2010).

### Data source

We performed an exhaustive literature search for all the factors that are involved in the cascade pathway in Medline (http://www.nlm.nih.gov/bsd/pmresources.html) and PubMed (http://www.ncbi.nlm.nih.gov/pubmed), by applying the search strings “Gene Name” and “Gene Name associated mutations”. A literature search was performed using the HGNC gene name. References listed in HGMD (http://www.hgmd.cf.ac.uk/ac/index.php) and UniProt (http://www.uniprot.org/) were cross-checked for the associated variants in coagulation disorders with reviewed publications. Medline contains indexed abstracts about coagulation factors. The OMIM provides curated literature information collected from the public accessed literature as well as other databases. Information related to a gene associated SAPs (rsIDs) was extracted from dbSNP (https://www.ncbi.nlm.nih.gov/SNP/) and counter-checked with UniProt (variants) to maintain consistency. The inclusion of SAPs information not only provides an overview of gene associated polymorphisms, but also provides the pathogenicity prediction scores for each SAP using various computational prediction methods.

### Database structuring

The CoagVDb is composed of five sections namely gene information, variant information, sequence information, structure information, and tool prediction (Figure 1). The first section ‘gene information’ includes genes coding the factors, gene IDs, gene symbol, taxon IDs, nucleotide sequence, chromosome location, epigenetic information, and map viewer. The second section ‘variants information’ consists of FASTA sequence, rs/Variant IDs, amino acid change and amino acid position. The third section ‘sequence information’ composed of amino acid sequence, sequence length, amino acid composition, ordered and disordered regions, disulfide bond composition, secondary structure characteristics and solvent accessibility. The fourth section ‘structure information’ provides information about the protein sequence IDs (NP), sequence length, and three-dimensional structures (3D). Also, we have included the information related to 3D coordinates such as experimental methods (NMR or X-Ray), resolution, chain type, and positions along with published literature. Last section ‘tool prediction’ contains prediction scores obtained for sequence and structure-based computational methods such as SIFT, PolyPhen 2, I-Mutant 3, fathmm, Align GVGD, PhD-SNP, SNPs&GO and SNAP to classify a variant as pathogenic and neutral.

## Additional file

Additional file 1. Table S1. Coagulation factor deficiencies and their prevalence in global population. Table S2. Detailed information regarding computational methods employed in classifying SAPs as disease or neutral.
