# Supplementary material for: CoagVDb: a comprehensive database for coagulation factors and their associated SAPs
Source: Biol Res. 2015 Jul 19;48(1):35. doi: 10.1186/s40659-015-0028-5 (PMC4506595; doi:10.1186/s40659-015-0028-5)
Supplement: Additional file 1. — Table S1. Coagulation factor deficiencies and their prevalence in global population. Table S2. Detailed information regarding computational methods employed in classifying SAPs as disease or neutral. [file 40659_2015_28_MOESM1_ESM.docx]

CoagVDb: A comprehensive database for coagulation factors and their associated SAPs

^1^Shabana kouser Ali, ^1,2^ *C. George Priya Doss, ^1^D. Thirumal Kumar,

^2^Hailong Zhu**

**Table S1.** Coagulation factor deficiencies and their prevalence in global population.

| **Factor** | **Common name** | **Bleeding Disorder** | **Mode of Inheritance** | **Prevalence** |
| --- | --- | --- | --- | --- |
| I | Fibrinogen | Fibrinogen deficiency   - Afibrinogenemia - Hypofibrinogenemia - Dysfibrinogenemia | Autosomal recessive    Autosomal dominant or recessive    Autosomal dominant or recessive | Afibrinogenemia is 1 in 1,000,000. Hypofib-rinogenemia and dysfibrinogenemia are more frequent than afibrinogenemia |
| II | Prothrombin | Prothrombin deficiency | Autosomal recessive | 1 in 2 million individuals |
| V | Proaccelerin | Proaccelerin deficiency | Autosomal recessive | Less than 1 in 1,000,000 |
| VII | Proconvertin | Proconvertin deficiency | Autosomal recessive | 1 in 500,000 |
| VIII | Anti-Hemophilic factor | Anti-Hemophilic factor deficiency | X-linked recessive | 1 in 5000 male births |
| IX | Christmas factor | Christmas factor deficiency | X-linked recessive | 1 in 30,000 male births |
| X | Stuart factor | Stuart factor deficiency | Autosomal recessive | 1 in 500,000 to 1,000,000 individuals |
| XI | Plasma thromboplastin  antecedent | Factor XI deficiency | Autosomal recessive | Incidence of 1 in 450 in Ashkenazi Jewish populations and 1 in 1,000,000 in non-Jewish populations |
| XII | Hageman factor | Hageman factor deficiency | Autosomal recessive | 1 in 1 million |
| XIII | Fibrin stabilizing factor | Factor XIII deficiency | Autosomal recessive | 1 in 2-5 million births |

**Table S2.** Detailed information regarding computational methods employed in classifying SAPs as disease or neutral

| **Computational Methods** | **INPUT** | **OUTPUT-Disease** | **OUTPUT-Neutral** | **Related web links** |
| --- | --- | --- | --- | --- |
| **SIFT** | NCBI GI number or RefSeq ID: | ≤0.05 | >0.05 | http://sift.jcvi.org/www/SIFT_BLink_submit.html |
| **PolyPhen2** | Protein FASTA sequence, substitution position (sequence residue number), native and new (mutant) residue (Single letter amino acid code) | 0.15-1 | 0.00-0.14 | http://genetics.bwh.harvard.edu/pph2/ |
| **IMutant3** | Protein FASTA sequence or Swiss-Prot protein code, substitution position (sequence residue number) and new residue (Single letter amino acid code) | Decrease:-0.5< Kcal/mol | Increase: 0.5>Kcal/mol | http://gpcr2.biocomp.unibo.it/cgi/predictors/I-Mutant3.0/I-Mutant3.0.cgi |
| **SNAP** | Protein FASTA sequence, substitution position (sequence residue number) and new residue (Single letter amino acid code) | Non-Neutral-Strongly predicted to change function | Strongly predicted as neutral | https://www.rostlab.org/services/snap/ |
| **SNPs&GO** | Protein FASTA sequence, substitution position (sequence residue number), and Wild and new residue after mutation (Single letter amino acid code) eg. WT+POS+NEW | Disease associated polymorphism (>0.5) | Neutral polymorphism | http://snps.biofold.org/snps-and-go/snps-and-go.html |
| **Align GVGD** | Multiple sequence alignment, substitution position (sequence residue number), and Wild and new residue after mutation (Single letter amino acid code) e.g. WT+POS+NEW | C55-C65-Substitutions most likely to interfere with function | C0-substitutions less likely to compromise function | http://agvgd.iarc.fr/agvgd_input.php |
| **PhD-SNP** | Protein FASTA sequence or Swiss-Prot protein code, substitution position (sequence residue number) and new residue (Single letter amino acid code) | Disease associated polymorphism | Neutral polymorphism | http://snps.biofold.org/phd-snp/phd-snp.html |
| **fathmm** | SwissProt/TrEMBL, RefSeq and Ensembl protein identifiers, substitution position (sequence residue number), and Wild and new residue after mutation (Single letter amino acid code) eg. WT+POS+NEW | Damaging | Tolerated | http://fathmm.biocompute.org.uk/inherited.html |
